# A fragment activity assay reveals the key residues of TBC1D15 GTPase-activating protein (GAP) in *Chiloscyllium plagiosum*

**DOI:** 10.1186/s12867-019-0122-2

**Published:** 2019-02-12

**Authors:** Yangyang Jin, Guodong Lin, Yanna Chen, Yinghua Ge, Ruofeng Liang, Jia Wu, Jianqing Chen, Dan Wang, Hengbo Shi, Hui Fei, Zhengbing Lv

**Affiliations:** 1 Zhejiang Provincial Key Laboratory of Silkworm Bioreactor and Biomedicine, College of Life Sciences, Zhejiang Sci-Tech University, Hangzhou, 310018 China; 2 The Hospital of Zhejiang Sci-Tech University, Zhejiang Sci-Tech University, Hangzhou, 310018 China

**Keywords:** TBC1D15, Rab-GAP activity, Evolution, Substrate specificity

## Abstract

**Background:**

GTPase-activating proteins (GAPs) with a TBC (Tre-2/Bub2/Cdc16) domain architecture serve as negative regulators of Rab GTPases. The related crystal structure has been studied and reported by other members of our research group in 2017 (Chen et al. in Protein Sci 26(4):834–846, 2017). The protein crystal structure and sequencing data accession numbers in Protein structure database (PDB) are 5TUB (Shark TBC1D15 GAP) and 5TUC (Sus TBC1D15 GAP), respectively. In this paper, we analyzed the Rab-GAP specificity of TBC1D15 in the evolution and influence of key amino acid residue mutations on Rab-GAP activity.

**Results:**

Sequence alignment showed that five arginine residues of the TBC1D15-GAP domain are conserved among the species *Sus/Mus/Homo* but have been replaced by glycine or lysine in Shark. A fragment activity assay was conducted by altering the five residues of Shark TBC1D15-GAP to arginine, and the corresponding arginine in TBC1D15 GAP domains from *Sus* and *Homo* species were mutated to resemble Shark TBC1D15-GAP. Our data revealed that the residues of G28, K45, K119, K122 and K221 in the Shark TBC1D15-GAP domain had a key role in determining the specificity for Rab7 and Rab11. Mutation of the five residues significantly altered the Shark TBC1D15-GAP activity.

**Conclusions:**

These results revealed that the substrate specificity of TBC1D15 has had different mechanisms across the evolution of species from lower-cartilaginous fish to higher mammals. Collectively, the data support a different mechanism of Shark TBC1D15-GAP in substrate selection, which provides a new idea for the development of Marine drugs.

## Background

Rab GTPases serve as major control elements in the coordination and definition of specific trafficking steps and intracellular compartments. Rab activity is modulated in part by GTPase-activating proteins (GAPs), which share a Tre-2/Bub2/Cdc16 (TBC) domain architecture [1]. The TBC domain is thought to be the product of the tre-2 oncogene and homologous to the yeast regulators of mitosis, Bub2 and Cdc16 [2]. With approximately 200 amino acids, the TBC domain is conserved among all eukaryotic cells [3]. Numerous studies have shown that TBC Rab-GAPs are important contributors to pathological states and that the mutation of key residues leads to disease [4–6]. For example, mutations of *Homo sapiens* TBC1D23 make it unable to exert its inhibitory effect on innate immunity signaling in spatiotemporal fashion, which contribute to a variety of diseases including sepsis, Crohn’s disease, atherosclerosis and cancer [7], and a TBC1D20 mutation is associated with Warburg Micro syndrome 4 (WARBM4), an autosomal recessive disorder characterized by congenital eye, brain, and genital abnormalities [8].

As a member of the TBC domain-containing proteins, TBC1D15 (TBC domain family, member 15) includes six motifs (A–F) with highly conserved arginines, such as Arg 245 on motif A and Arg 400 on motif B, which are associated with Rab-GAP activity [9]. TBC1D15 plays an important role in mitochondrial morphology [10] and stem cell self-renewal [11, 12]. TBC1D15 protein is crucial for the proper localization of Rho A during the formation of cytoplasmic division rings [13]. In combination with mVps39, TBC1D15 influences lysosomal morphology and retains growth factor dependence [14]. Collectively, these findings reveal an important role of TBC1D15 in signal transactivation. Our data in *Chiloscyllium plagiosum* showed that TBC1D15 may be associated with diabetes due to the active peptide at its N-terminus, which has immunomodulatory and inhibitory activity on lipid peroxidation [15]. However, unlike its roles in humans and rodents, the roles of TBC1D15 in marine organisms remain unclear.

Recent data from *Chiloscyllium plagiosum* revealed that TBC1D15 had Rab-GAP activity for Rab7a [16] and Rab11a [15]. Considering the role of TBC1D15 in species evolution [17, 18] and the difference of some arginine residues between *Chiloscyllium plagiosum* and human [15], we hypothesize that the arginine residues in Shark TBC1D15 play a crucial role in its functions. In this study, to evaluate the role of arginine residues in TBC1D15 of *Chiloscyllium plagiosum*, we mutated and purified TBC1D15-GAP proteins of shark, *Sus* and *Homo* species, respectively. Five key arginine residues affecting the activity of TBC1D15-GAP protein in sharks were revealed by the activity measurement before and after the mutation.

## Results

### Amino acids sequence analysis of TBC1D15-GAP

**Fig. 1:**
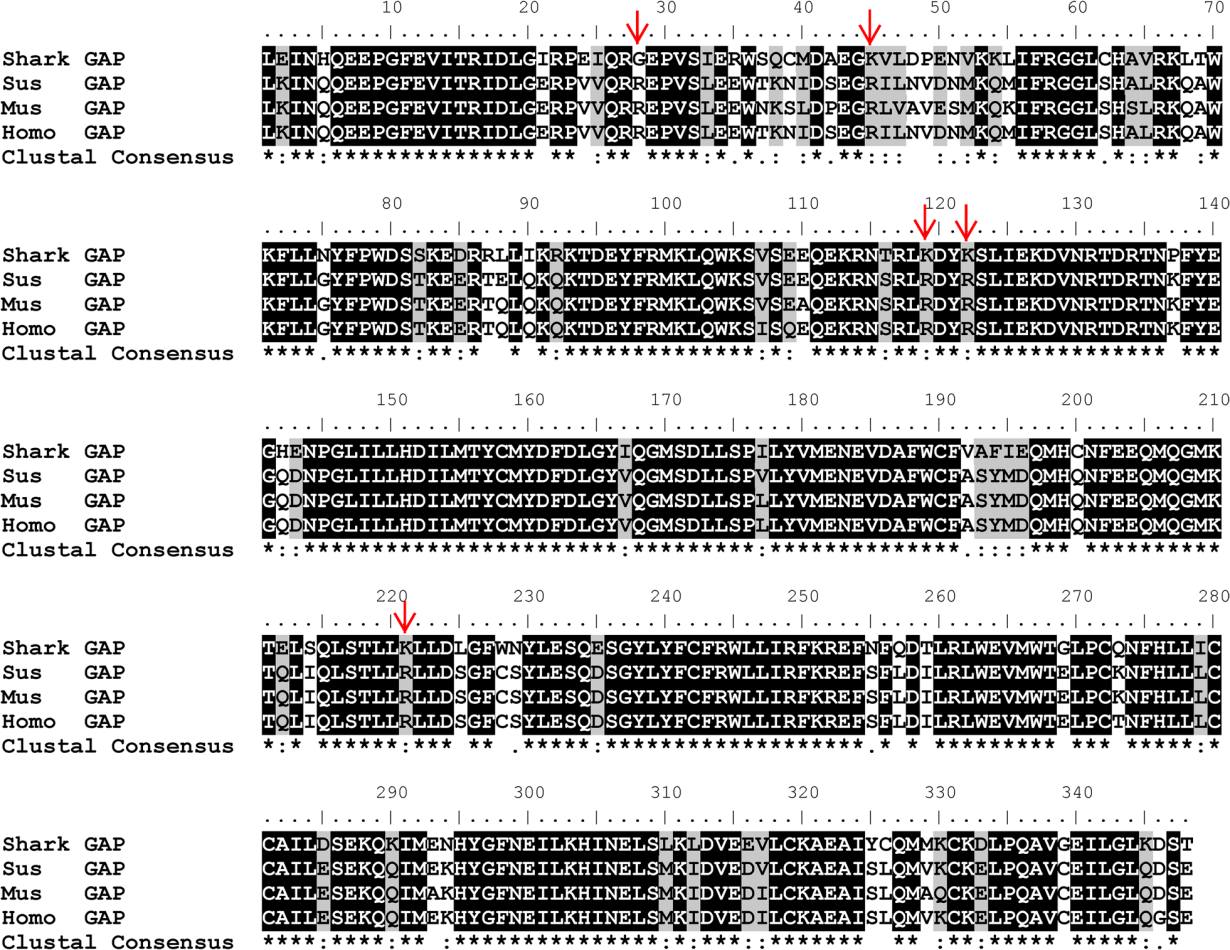
Conservation analysis of TBC1D15-GAP sequences of different species. Conservation analysis was conducted with the BioEdit program, and conserved amino acids are shown in red. The arrows indicate arginine residues that are conserved in (Sus, Mus, and Homo) GAPs but replaced by glycine or lysine in Shark TBC1D15-GAP

The sequence alignment showed that TBC1D15-GAP is conserved across the studied species (Fig. 1). It is worth noting that the residues of the five sites indicated by the arrow are G28, K45, K119, K122 and K221 in the shark, respectively, while all are arginine in *Sus*, *Mus* and *Homo*.

### Purification of the TBC1D15-GAP domain and Rab proteins

**Fig. 2:**
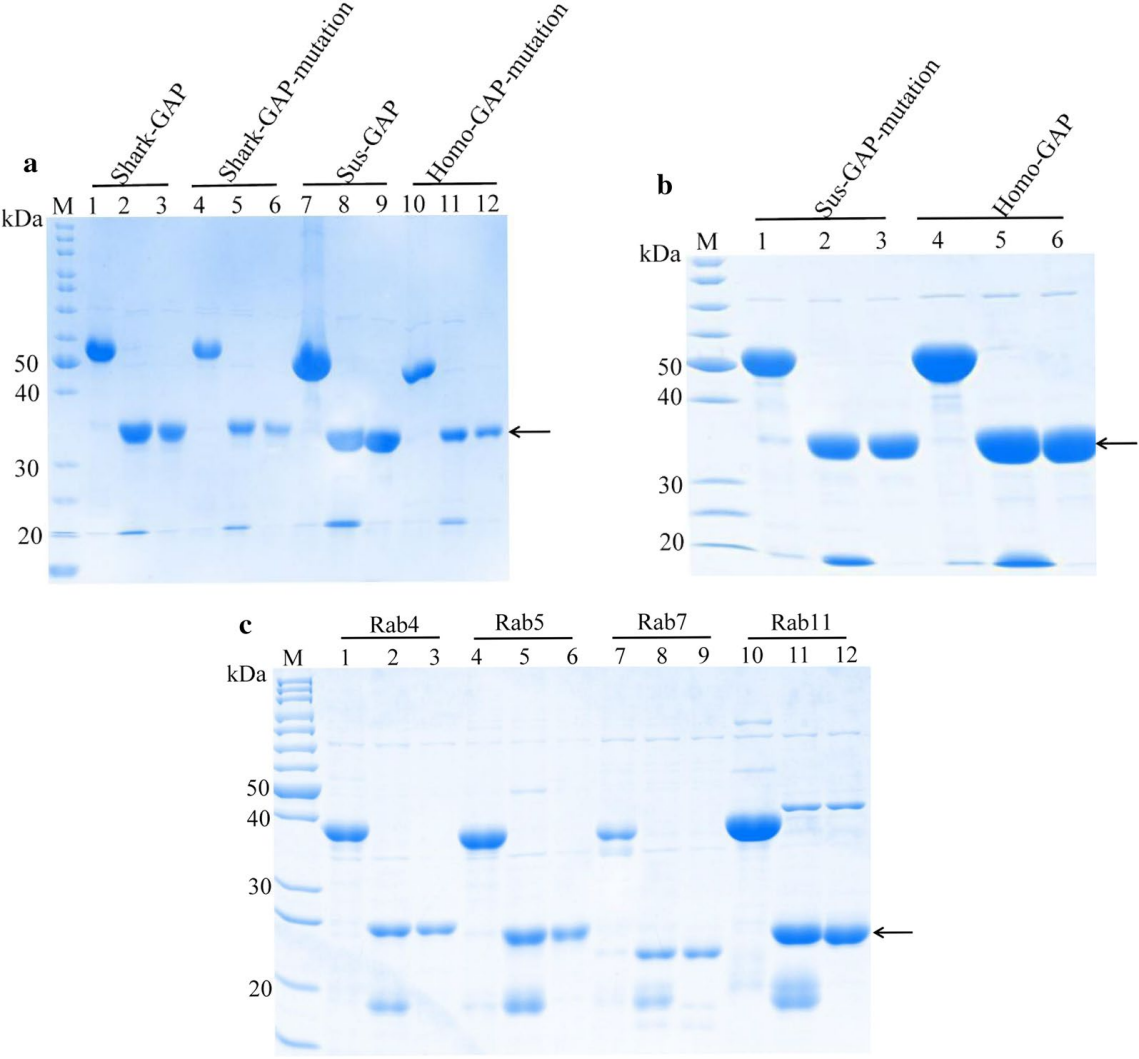
Protein expression and purification. Proteins were analyzed by SDS-PAGE. Lane 1, 4, 7, 10 displays the proteins with a His-sumo tag. Lane 2, 5, 8, 11 shows the proteins that were cut by the Ulp1 enzyme. Lane 3, 6, 9, 12 displays the purified proteins. **a** Shark-TBC1D15-GAP/mutation, Sus-TBC1D15-GAP and Homo-TBC1D15-GAP-mutation. **b** Sus-TBC1D15-GAP-mutation and Homo-TBC1D15-GAP. **c** Rab4/5/7/11

Rab-GAP activity is determined by the GAP domain of TBC1D15 [19–21]. The fragments containing Shark-, Sus- or Homo-TBC1D15-GAP domain were inserted into a plasmid and expressed in *E. coli* BL21 (DE3). The processes of protein expression and purification were performed as described previously [15]. Generally, after expression, the proteins were purified by Superdex 200 gel filtration chromatography (320 mL). We collected the largest peak of the elution profile and conducted SDS-PAGE. As is shown in Fig. 2a and b, the bands from wild type or mutated Shark-, Sus- or Homo-TBC1D15-GAP correspond to a molecular mass of 38 kDa. After that, the Rab proteins were also expressed and purified (Fig. 2c).

### GAP assays of Shark/Sus/Homo-TBC1D15-GAP

**Fig. 3:**
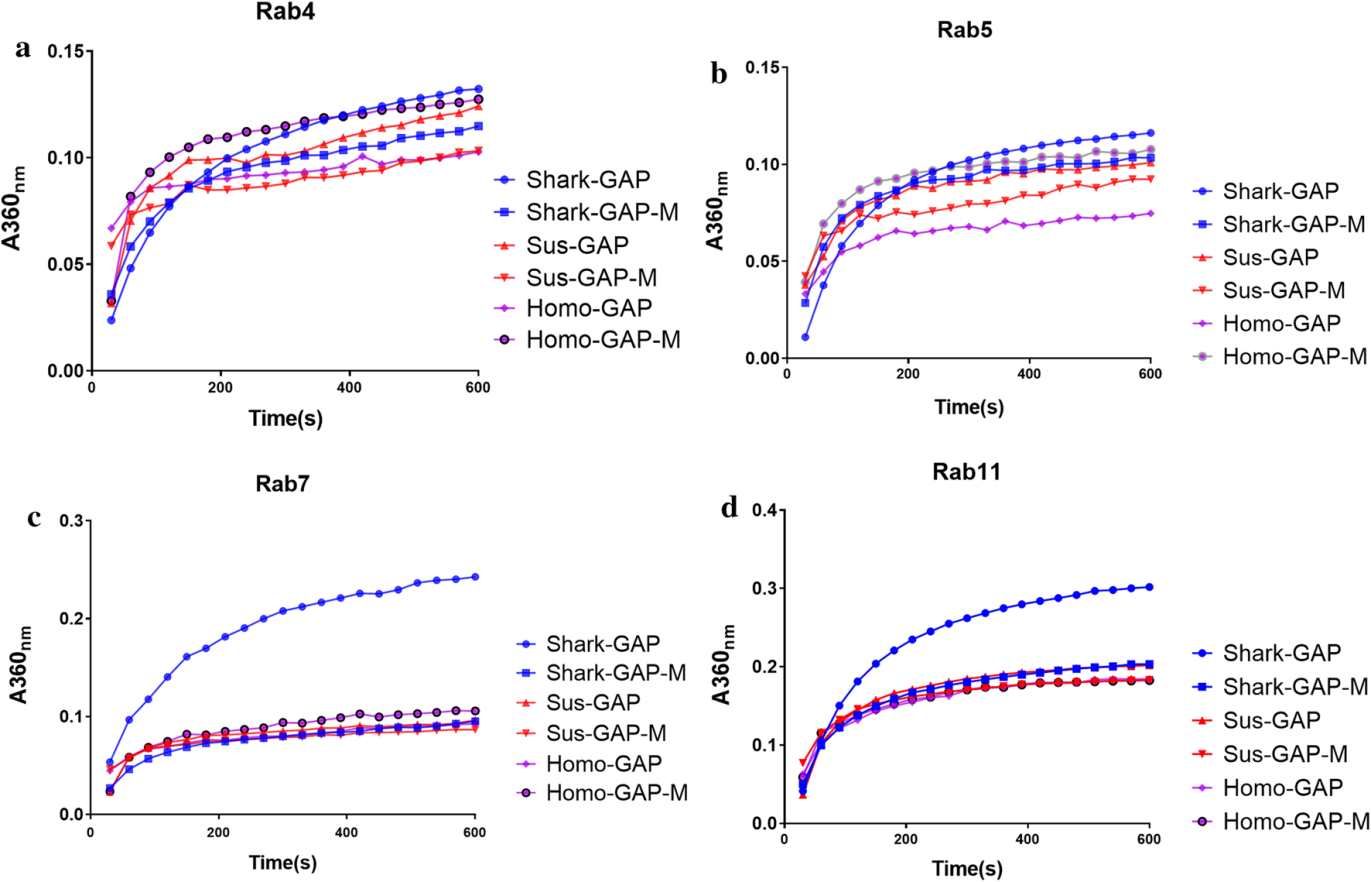
The kinetic analysis of Rab-GAP activity on Shark/Sus/Homo-TBC1D15-GAP/GAP mutants. In vitro GAP assay of Shark/Sus/Homo-TBC1D15-GAP/GAP mutants using Rab4/5/7/11 as the substrate

A Rab-GAP activity assay was conducted via analyzing the hydrolysis rate of Rab coupled with GAP proteins. As is shown in Fig. 3a, the TBC1D15-GAP of Shark, *Sus* and *Homo* have a low catalytic efficiency for Rab4. Mutation of Shark or *Sus* TBC1D15-GAP led to a lower catalytic efficiency for Rab4, whereas mutation of *Homo* TBC1D15-GAP increased its catalytic efficiency. The mutation of Shark TBC1D15-GAP decreased the catalytic efficiency for Rab5 which was about the same as that of *Sus* TBC1D15-GAP. The arginine mutation of *Homo* TBC1D15-GAP increased the catalytic efficiency for Rab5. The mutation of *Homo* TBC1D15-GAP had a similar catalytic efficiency as Shark TBC1D15-GAP (Fig. 3b). Regarding the catalytic efficiency of TBC1D15-GAP for Rab5 and Rab4, that of Shark had the highest efficiency, followed by *Sus* and *Homo*.

As is shown in Fig. 3c, Shark TBC1D15-GAP had the highest catalytic efficiency for Rab7 among the TBC1D15-GAPs of the three species. Mutation of Shark TBC1D15-GAP decreased the catalytic efficiency for Rab7 by half. Wild-type *Homo* and *Sus* TBC1D15-GAP had similar catalytic efficiencies for Rab7, whereas their mutations demonstrated no significant change. Shark TBC1D15-GAP had a higher catalytic efficiency for Rab11 than for Rab7. Shark-mut had a lower catalytic efficiency for Rab11, which was equivalent with that of *Homo* and *Sus*. However, the catalytic efficiency of Homo-mut and Sus-mut for Rab11 was rescued (Fig. 3d).

### Substrate-specificity analysis of TBC1D15-GAP and their mutants

In this study, the catalytic efficiency parameter k_cat_/K_m_ was calculated to assess the Rab-GAP activity of Shark/Sus/Homo-TBC1D15-GAP and their mutants. As is shown in Table 1, Shark-GAP had the highest specificity with Rab5, followed by Rab4, Rab11 and Rab7. An amino acid mutation of Shark-GAP (Shark-GAP-mut) altered the Rab specificity and led to a catalytic specificity for Rab11. Both Sus-GAP and Homo-GAP had a high catalytic specificity for Rab11. However, their mutations had no significant effect on catalytic specificity for Rabs.

**Table 1:** The catalytic efficiency parameter (k_cat_/K_m_) values of Shark-/Sus-/Homo-TBC1D15-GAP/GAP mutants

Fragments	Rab4	Rab5	Rab7	Rab11
Shark-GAP	113.8 ± 20.5^bA^	212.8 ± 43.8^aA^	90.9 ± 20.4^bcA^	94.8 ± 13.5^bcB^
Shark-GAP-mut	52.8 ± 10.5^bB^	56.1 ± 4.78^bB^	53.3 ± 9.47^bB^	130.6 ± 20.5^aA^
Sus-GAP	56.9 ± 24.7^bB^	72 ± 13.6^abB^	56.3 ± 17.5^bB^	105 ± 26.6a^B^
Sus-GAP-mut	39.9 ± l7.02^bB^	57.6 ± 12.5^bB^	56.3 ± 6.18^bB^	105 ± l 7.4^aB^
Homo-GAP	60.6 ± 16.l3^aB^	69.7 ± 17.63^aB^	55.1 ± 9.47^aB^	74.9 ± 15.7^aB^
Homo-GAP-mut	68.3 ± 16.5^aB^	61.1 ± 16.4^aB^	55.6 ± 5.3^aB^	66.8 ± 27.5^aB^

The results showed above are mean values ± SD from three independent experiments. Different uppercase letters indicate significant (P < 0.05) differences among values within a column. Different lowercase letters indicate significant (P < 0.05) differences among values in a row

Shark-GAP had the highest catalytic specificity for Rab4, Rab5 and Rab7 among the GAPs of the three species. Shark-GAP-mut had significantly decreased catalytic specificity, which was confirmed by decreased catalytic efficiency parameter values of k_cat_/K_m_ for Rab4, Rab5 and Rab7. Shark-GAP-mut had k_cat_/K_m_ value similar to those of Homo- and Sus-GAP for Rab4, Rab5 and Rab7. However, relative to Shark-GAP, Shark-GAP-mut had increased catalytic efficiency for Rab11, which was similar to that of Homo- and Sus-GAP and their mutations.

## Discussion

TBC1D15 participates in several regulatory networks. Our recent data in vitro suggest that Shark-TBC1D15 exhibits Rab-GAP activity on Rab7 [16] and Rab11 [15]. At the N-terminus of Shark-TBC1D15, there is an APSL (active peptide from Shark liver) domain, which is involved in liver regeneration in *Chiloscyllium plagiosum* [22]. APSL is suggested to be associated with reducing blood glucose levels in mice with type 2 diabetes. By analyzing the structure of Shark TBC1D15, it is proved that arginine and glutamine on the catalytic active site are the key catalytic amino acids, and any mutation of them will lead to the loss of GAP activity [15]. We selected to mutate highly conserved arginine residues and analyzed the activity and substrate specificity of TBC1D15-GAP in different species. Our data confirmed the previous hypothesis [15].

It was speculated from the GAP assays that the effect of five specific arginine residues of TBC1D15 on the activity of Rab-GAP might be related to the evolutionary process of species from low-cartilage fishes (*Chiloscyllium plagiosum*) to higher mammals (*Homo* sapiens). This provides a new method for species identification. The high catalytic efficiency of Shark-GAP for Rab7 and Rab11 is consistent with our previous data showing that Rab7 and Rab11 are substrates of Shark TBC1D15 [15, 16]. The catalytic efficiency of Shark-GAP-mut on Rab7 was significantly reduced compared with Shark-GAP, suggesting that the mutated 5 amino acid sites play a key role in the function of Shark TBC1D15. However, Sus- or Homo-GAP-mut did not rescue its catalytic efficiency for Rab7, suggesting that the reaction mechanism of Sus- and Homo-GAP is different from that of sharks. We speculate that there are other residues that control the activity of Sus- and Homo-GAP. The truncated proteins of Shark/Sus/Homo-TBC1D15 all have Rab-GAP activity. The highly evolved *Sus* and *Homo* species have a similar preference for Rab4/5/7/11 substrates, and both have the greatest substrate preference for Rab11. The wild-type Shark-TBC1D15-GAP has a high substrate preference for Rab5, but when the residues G28, K45, K119, K122 and K221 are mutated to arginine, Rab11 becomes the optimum substrate, which is similar to Sus/Homo-TBC1D15-GAP. In addition, a mutant of Homo-TBC1D15-GAP has a high substrate preference for Rab4. The results further demonstrated the important role of the residues G28, K45, K119, K122 and K221 in Rab-GAP activity during the evolution from sharks to humans. Therefore, the substrate-specificity analysis indicated that the mutation of conserved amino acids can influence the activity and specificity of Rab-GAP in different species. This lays the foundation for studying the mechanism of related diseases and has guiding significance for the target selection of future new drug research and development.

It can be seen from the above analysis that in low-grade cartilaginous fishes, TBC1D15 can select a variety of Rab substrates, and the difference in preference is not obvious. With the continuous evolution of species, the preference of TBC1D15 tends to select Rab11, which is also a reflection of the increasing adaptability of species to the environment. On the other hand, we found that the expression level of GAP protein decreased significantly after the mutation of specific amino acids, which indicating that these amino acid sites play an crucial role in the transcription, translation and other expression processes of the protein. Because the TBC1D15 protein is associated with a variety of diseases, eg. Alzheimer’s disease, Type 2 diabetes and cancer. Therefore, it is speculated that these amino acid sites may be related to the regulation of some diseases. Besides, it provides a certain reference value for the future study on the related functions of TBC1D15 and other TBC family proteins. Think about it the other way, functional changes or abnormalities in GAP proteins may cause functional changes or abnormalities in Rab proteins. Rab proteins are involved in many processes of membrane vesicle transport and can mediate the trafficking of proteins, so it plays a very important role in life activities. It’s worth noting that Rab proteins are closely related to the occurrence and development of certain tumors. For example, Rab25 expression in breast cancer tissues is 67% higher than that in normal breast epithelial cells [23]. The Rab protein also plays an important role in the normal development of the nervous system. Therefore, this experiment provides a broad idea for the study of Rab protein-related diseases. We believe that both GAP and Rab proteins may become one of the targets for the treatment of diseases and provide new treatment schemes for related diseases.

## Conclusions

In conclusion, the results suggest that the substrate specificity of TBC1D15 has different mechanisms across the evolution of species from lower-cartilaginous fish to higher mammals. Our data revealed that the residues G28, K45, K119, K122 and K221 in the Shark TBC1D15-GAP domain play a key role in regulating specificity for Rab7 and Rab11. These residues also affect Shark TBC1D15-GAP activity. However, the reason why TBC1D15 has specificity for Rab11 and Rab7 and the mechanism by which it plays a role in signal trafficking in Shark liver require exploration. Collectively, the data indicate that TBC1D15 has a different mechanism of substrate selection in Shark TBC1D15-GAP from in that of *Sus* and *Homo*, which imitates the function of Shark TBC1D15-GAP in marine drugs development.

## Methods

### Materials

The *E. coli* strains TG1 and BL21 (DE3); the plasmids pETduet-His-sumo-Shark-TBC1D15-GAP, pETduet-His-sumo-Sus-TBC1D15-GAP, and pETduet-His-sumo-Homo-TBC1D15-GAP; and the vectors pETduet-His-sumo-Rab4/Rab5/Rab7/Rab11 were gifted from Professor Jianhong Shu of Zhejiang Sci-Tech University. A Plasmid Mini Kit, Agarose Gel Extraction Kit and PCR Clean Kit were purchased from Axyen (Beijing, China). Restriction enzymes, T4 DNA ligase and DNA markers were obtained from Takara (Beijing, China). KOD-plus DNA Polymerase was purchased from TOYOBO (Shanghai, China). Protein marker was obtained from Thermo Fisher Scientific (Shanghai, China). GTP powder was obtained from Aladdin (Shanghai, China). Ni–NTA beads and 50 ml gravity columns were obtained from GE Healthcare (Beijing, China).

### Sequence analysis of TBC1D15-GAP proteins

The BioEdit program was used to analyze the conservation of TBC1D15-GAP amino acid sequences from four species (Shark, *Sus*, *Mus*, *Homo*).

### Recombinant protein expression and purification

Plasmids of pETduet His-sumo-TBC1D15-GAPs mutants (Shark-, Sus-, Mus-, Homo-GAP-mut) were synthesized by GENEWIZ (Suzhou, China). Rab proteins were purified following the same protocol, and an extra 10 mM MgCl_2_ was added in each step. Later, the protein concentration was determined by the Qubit^®^ Protein Assay Kit, and Rab proteins were incubated with a 25-fold molar excess of GTP on ice for 2–3 h. A desalting column (GE Healthcare Life Science) was used to remove the excess GTP, and the Rab-GTP complexes were concentrated to 4-8 mg/mL, then placed it in liquid nitrogen for quick freezing, and stored at − 80 °C until analysis.

### GAP assays

GAP-accelerated GTP hydrolysis was measured using the EnzChek Phosphate Assay Kit (Invitrogen). In total, 25 μM Rab-GTP complexes were mixed with 5 μM GAPs and loaded in 96-well plates containing 50 mM Tris pH 7.5, 10 mM MgCl2, 0.2 mM 2-amino-6-mercapto-7-methylpurine riboside (MESG), and 1 U/ml of purine nucleoside phosphorylase. Phosphate (Pi) production was recorded as the change in absorbance at 360 nm using a Multiscan spectrometer (SpectraMax 190, Molecular Devices) every 30 s for up to 10 min. Data were analyzed using a time-course curve.

### Standard curve for inorganic phosphate

The phosphate standard solution was diluted 100-fold with ddH_2_O and then it was used to prepare 500 M phosphate standard working solution, and the linear range of the assay for Pi extended from 0 to 150 μM. Variable amounts of the phosphate standard working solution were added to the standard reaction mixtures and incubated for 30 min at 22 °C.

### The activity of TBC1D15-GAPs and mutants on Rabs

The concentrations of TBC1D15-GAPs and their mutants were measured, concentrated to 0.25 μM, and mixed with various concentrations of Rab-GTP complexes (0, 10, 20, 30, 40, 60, 80, and 100 μM). Each assay was performed in triplicate, and the amount of Pi released was assessed from the corresponding values obtained from a standard curve. Data were analyzed by fitting them simultaneously to the pseudo-first-order Michaelis–Menten model function. The catalytic efficiency (k_cat_/K_m_) and intrinsic rate constant for GTP hydrolysis (kintr) were treated as global parameters [24].

## Abbreviations

GAP(s): GTPase-activating protein(s)
TBC: Tre-2/Bub2/Cdc16
TBC1D15: TBC domain family, member 15
Arg: arginine
APSL: active peptide from Shark liver
